# An Introduction to Health Literacy and Social Contexts with Recommendations for Health Professionals and Researchers

**DOI:** 10.3390/ijerph21020240

**Published:** 2024-02-19

**Authors:** Joy Agner, Katharine Elizabeth Bau, Dirk Bruland

**Affiliations:** 1 Chan Division of Occupational Science and Occupational Therapy, University of Southern California, Los Angeles, CA 90089, USA; kbau@usc.edu; 2 Institute for Educational and Health-Care Research in the Health Sector, Bielefeld University of Applied Sciences and Arts, Interaktion 1, 33619 Bielefeld, Germany; dirk.bruland@fh-bielefeld.de

## 1. Introduction

Rarely do individuals seek, obtain, and understand health information in a solitary void. Nevertheless, most research treats health literacy as an individual-level construct. Individual conceptualization and measurement of health literacy can limit health literacy interventions and theory by ignoring how social contexts define, shape and influence how health information is accessed and understood. We aim to address this research gap by examining the multifaceted ways in which social contexts influence health literacy. We link cutting-edge research on social contexts and health literacy with extant literature by summarizing eleven articles for the special issue, using research traditions identified in this area. Author teams represented seven countries and examined social influences on health literacy in diverse contexts including heath care settings, community-based mental health centers (Clubhouses), sheltered workshops, universities, libraries, digital spaces, and others. In addition to diversity in geography and setting, these 11 articles consider unique social factors influencing health literacy for various populations including university students, children, individuals with intellectual disabilities, individuals with mental illness, among others. We conclude with recommendations for health professionals and researchers. These recommendations revolve around four main themes: (1) the need for a comprehensive, multi-level intervention framework to guide practice and research; (2) strategies to leverage natural social contexts and resources to enhance health and health literacy in vulnerable populations; (3) the increasing necessity to focus on digital interaction spaces and online communication (both true and false information) to address health literacy gaps; and (4) bidirectional influences between improving community health and health literacy.

### 1.1. Background on Health Literacy in Social Contexts

We increasingly understand the ways in which health literacy—our ability to find, seek, and utilize health information [1,2,3,4]—is deeply shaped by our social contexts [5,6]. The social environment and the culture we are living in implicitly and explicitly influence our health-related knowledge and, in turn, our health decisions [7,8,9,10]. Individual factors, such as education, age, race, cognitive and reasoning abilities, language fluency, and familiarity with medical terminology, determine an individual’s health literacy [11,12,13,14,15,16,17]. However, as exemplified in an anecdote below, individual factors intersect and interact with social contexts and social capital to influence and inform our health knowledge and decision making [18,19,20,21]. 

For example, last year, a family member of one of the authors of this paper was diagnosed with prostate cancer. Based on her background, she offered to help him understand available provider options, strategies for accessing his personal health information (such as lab tests and visit summaries) and interpret the health information he received to make treatment decisions. Knowing that the healthcare in the rural area where he lived might be lacking, she was able to find a cancer researcher who specialized in his diagnosis for a second opinion. Although she was in a different state and could not attend his appointments, he and his wife audio-recorded them for her, allowing the group to discuss his options together and write out follow-up questions for his doctor. After receiving the healthcare professionals’ advice on diverse treatment options, she searched the literature to compare the published efficacy of these approaches and outcomes based on his age group. She was able to present the options in layman’s terms and discuss the pros and cons with him based on what she knew of his personal values. He also consulted and relied on her sister, who is a nurse, and his wife, who he trusts deeply, to inform his decisions.

This illustrates how his ability to seek, obtain, and understand health information was not only based on his education, cognition, and individual values, but was also explicitly shaped by a social network that included his family, as well as the doctors and nurses that informed his care. Beyond those explicit interactions, she noticed through their conversations that his health knowledge and decision-making processes were influenced by the health organizations within which he received care, broader cultural values regarding cancer treatment options at different ages, and the online media that he consumed related to his diagnosis. There were likely other additional social influences impacting his health literacy and decision making in this situation beyond her awareness.

Upon introspection, many of us can relate to the ways in which social contexts have affected our own abilities to seek, obtain, and understand health-related information, and how others have relied upon us as well. However, research and theory often conceptualize health literacy as an individual construct. This myopic view of health literacy may limit our ability to understand the true context in which health information is accessed and understood [19,21]. Thus, the purpose of this Special Issue is to advance research and theory on the intersection of social contexts and health literacy. Better understanding the structure and function of social contexts in health literacy for diverse populations can advance theory, inform intervention development, and hopefully enhance our ability to leverage health literacy to improve health outcomes.

### 1.2. Definitions, Characteristics of Included Articles, and Conceptual Strategy

The dominant understanding of health literacy in the early days was closely associated with medicine, healthcare research, and the conventional use of the term “literacy”, i.e., patients’ reading and writing skills [22]. The American Medical Association, for example, writes that health literacy is “… the constellation of skills, including the ability to perform basic reading and numeral tasks required to function in the healthcare environment” [23]. Over the past 20 years, the concept of health literacy has expanded to more closely reflect the wide variety of skills needed to access and interpret health information and to navigate health services, and it has grown to include sub-definitions for unique skill sets. Basic numeracy and reading skills would be described as functional health literacy [22,24,25], while interactive health literacy considers social and cognitive skills [22,24,25]. Critical health literacy builds on the aforementioned skills and involves an approach to interactions with providers and health systems that recognizes power [26,27], patient choice, shared decision making [28], and social determinants of health [29]. Today, health literacy is commonly defined as “people’s knowledge, motivation and competences to access, understand, appraise, and apply health information in order to make judgements and decisions in everyday life concerning healthcare, disease prevention and health promotion to maintain or improve quality of life during the life course” [4]. Health literacy is increasingly being seen as a crucial factor for health-related outcomes, an aspect of empowerment [26,30] and an influencing factor related to health equity [31]. Health literacy has effects on health behaviors, healthcare usage, and, therefore, healthcare costs [32,33,34].

The understanding of the term has since changed further. In the WHO Shanghai Declaration in 2016, health literacy was stated as being more than the responsibility of a single individual [35]. Bronfenbrenner (2009) highlighted that the social environment directly or indirectly influences individuals to a high degree, including their behaviors, thoughts, and feelings [36]. Individuals are embedded in the social context [37,38]. However, the research on social contexts and health literacy is lacking, particularly in regard to quantitative and population-level studies [10]. This gap between theory and either conceptual or empirical research provides the rationale for this Special Issue, which aims to increase scholarship on health literacy and social contexts, with practical implications for clinicians and scholars.

In our solicitation for this Special Issue, we defined social contexts as interpersonal relationships, organizations, communities, health and educational systems, cultural contexts, and digital interaction spaces. We invited conceptual and theoretical work, empirical articles, and reviews that could expand people’s knowledge of how social contexts influence their access to health information, their interpretations of that information, and/or their health-related decision-making processes. In total, 11 articles were selected for publication after peer review. These are represented in Table 1. The author teams represented seven countries and examined the social influences on health literacy in diverse contexts, including heath care settings, community-based mental health centers (Clubhouses), sheltered workshops, universities, libraries, digital spaces, and others. In addition to being diverse in geography and setting, these 11 articles also considered unique social factors influencing health literacy for multiple populations, including university students, children, individuals with intellectual disabilities, and individuals with mental illness, among others.

**Figure 1:**
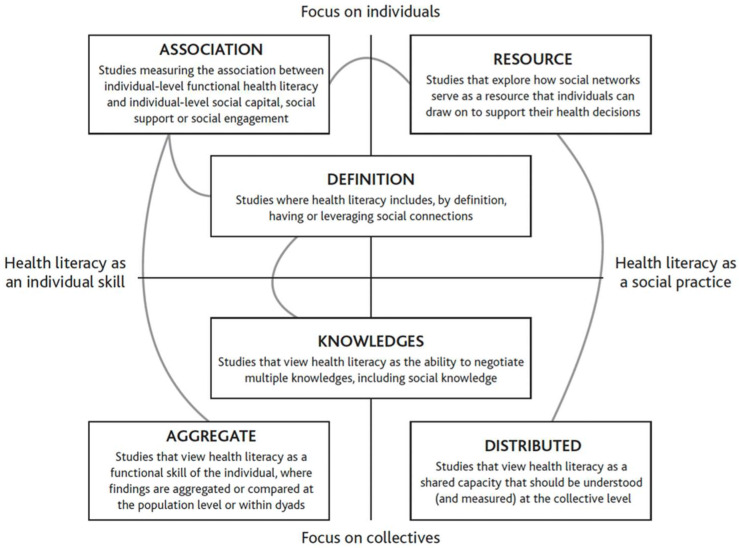
Health literacy and social context research traditions and definitions [50] (p. 672).

We consider the main findings from these articles using a conceptual strategy to understand health literacy and social contexts developed by Pitt et al. (2019) [50]. Pitt and colleagues conducted a meta-narrative review of health literacy in a social context to better understand its conceptualization, methodological diversity, insights drawn from this line of research, and theoretical clarity. They analyzed 53 qualitative and quantitative articles linking health literacy and social context and identified six “research traditions” which “should not be seen as separate streams of research, but as different channels of a braided river, splitting off and rejoining” (p. 668). We build on this work and contribute to these braids and channels (i.e., identified research traditions) to conceptualize the new work in this Special Issue. An image of how Pitt and colleague’s research traditions were conceptualized and how they link together is included Figure 1 for visual reference. These research traditions include association, resources, distributed, definition, aggregated, and knowledge. The meanings of these terms are included below and reiterated in context as they are applied to each article from the Special Issue.

Using this approach, we hope to both link cutting-edge research with existing traditions, and highlight continuing gaps in methods and theory. We conclude with overarching themes and provide suggestions for health professionals and researchers to apply these findings to their practice areas.

## 2. Health Literacy and Social Context Findings from the Special Issue

Studies highlighted in this Special Issue intersect with a broad diversity of research traditions on health literacy and social contexts. Vetter et al.’s (2022) [49] work contributed to the research tradition focused on *defining* health literacy in social contexts and did so for individuals with mild to moderate intellectual disabilities in Germany. Using 38 guided interviews, they found that health literacy is *defined and shaped* by multiple levels of social context, including educational settings, interpersonal relationships, organizational and social structures, the healthcare system, politics, cultural contexts, and digital interaction spaces.

These dimensions of social context highlight opportunities to address health literacy on structural rather than individual levels for people with intellectual disabilities. For instance, in the healthcare setting, there are multiple considerations that define the health literacy context such as whether information is offered in audio or visual formats, how much support exists for healthcare providers, and whether clear communication is utilized to make appointments or to relay health information. Interestingly, Vetter and colleagues noted the influence digital spaces can have on every level of social context, such as through social media and smartphone usage. However, they also noted that individuals with intellectual disabilities are often excluded from these digital spaces, creating a digital divide where individuals with intellectual disabilities do not gain the same benefits from expanding the digitization of health information. Their research points out the lack of interventions supporting health literacy for individuals with intellectual disabilities at contextual and organizational levels. In doing so, they highlight opportunities to identify different levels of social context that affect the abilities of people with mild to moderate intellectual disabilities to obtain and understand health information and opportunities to design interventions targeting those social contexts.

Like Vetter and colleagues, Dins and Keeley (2022) [42] explored the role of social connection and health literacy among individuals with intellectual disabilities, but they focused on a population with profound intellectual disabilities (PIDs), who may have limited ability to understand, or to communicate their understanding, of health-related information and engage in shared decision-making. Thus, Dins and Keeley examine the role of caregivers in addressing and communicating health-related needs among people with PIDs in Germany and how and whether individuals with PIDs can be engaged in their own health-related decision making. Using a multi-method Delphi study approach, they began by interviewing 14 experts with knowledge about people with PIDs, including people in academia, funding agencies, and service providers, and used those findings to develop an online survey for care professionals (*n* = 111), primarily those working daily with people with PIDs in sheltered workshops. This step of the research was followed by three ethnographic case studies of people with PIDs and their support networks, which were conducted using a diverse variety of creative strategies to match the communicative abilities of their participants.

The results of this staged, multi-method study highlight the importance of social context among individuals with PIDs, but they also stress that, even in the case of profound PIDs, individuals “want to and are able to participate in communication about health-related issues” (p. 11). Facilitating this communication may require creativity and deep knowledge of the communicative abilities of the individuals, as demonstrated by the researchers, who engaged people with PIDs in their research. Finally, the researchers examined system-wide healthcare strategies to enhance caregivers’ abilities and the perceived value of diverse communication strategies (text, video, face-to-face augmented communication, and others, including haptic and tactile strategies) to engage people with PIDs in health-related decision making. The importance of care partners being involved in health-related decisions and supporting patients in communicating health-related concepts was emphasized. Additionally, healthcare professionals were encouraged to adapt resources, use multisensory approaches, and utilize visual and symbolic information to convey health information. The study highlighted how health literacy should be responsive and individualized, and that this is possible for individuals with profound barriers to cognition and communication.

Unlike the study involving people with intellectual disabilities completed by Vetter, which related to the definitional research tradition, Dins and Keeley contributed new findings related to the *knowledge* tradition of health literacy in social context research. Within this research tradition, social skills are considered forms of knowledge used to access and obtain health information. Interestingly, this is typically conceived on behalf of the knowledge of the individual seeking health information, but in this case, Dins and Keeley examine, among other things, how *caregiver and provider knowledge* of how to interact socially with people with PIDs can limit or enhance their abilities to understand health-related information and engage in health-related decision making.

A more conventional study in the *knowledge* tradition of social health literacy research was offered by Larson and Gilstad (2022) [44]. Using a systematic review approach, Larson and Gilstad (2022) [44] analyzed qualitative studies focusing on how patients understand online health information and communicate their findings with healthcare professionals. Of the 16 studies included in their review, ten focused on the patient perspective, four addressed the provider perspective, and two articles included both perspectives. From the patient perspective, Larson and Gilstad found that individuals sought out health information online for a variety of reasons, including for self-diagnosis or to be more informed when attending medical visits. Higher socioeconomic groups were more proactive in seeking out health information online, which led to higher health literacy and health management, while lower socioeconomic groups took on more passive roles and often had paternalistic views of their providers. Some participants found online health information to support and supplement the advice received from the doctor, while others noted barriers in wanting to discuss online health information with their doctors due to embarrassment, uncertainty, or a perceived lack of knowledge. From the healthcare provider perspective, strategies for responding to patient questions about online health information were important, including helping patients contextualize online health information and pointing them towards reputable sources. This study highlights how multiple levels of social context intersect and interact. The digital interaction space, where health information was accessed, empowered patients to take an active role in their own health. But ideally, health information was also contextualized and appraised through social interactions with providers.

Like the study by Larson and Gilstad, Phillips et al.’s (2022) [47] research contributed to the *knowledge* tradition of social context and health literacy by examining how Hawai’i residents, aged 18–35, accessed health information from their social networks during the COVID-19 pandemic. Social knowledge and information about who to discuss health matters with and where to search for health information were crucial to their understanding of COVID-19 and its impact on health. The social behaviors around seeking health information seemed to differ based on perceived risk, with individuals reporting a greater perceived risk of contracting COVID-19 discussing their health with more individuals. Similar to Larson and Gilstad, Phillips and colleagues found that individuals rely on both online health information and personal interactions to seek and interpret health information.

Understanding the profound impact that digital interaction spaces have on understanding health behavior points to the need to prepare providers to assist patients in accessing and vetting online health information—also described as eHealth literacy. Naturally, this also requires providers to have a high level of eHealth literacy. Mather et al. (2022) [46] contributed to this line of research by examining work readiness and the perceived ability to support future patients in accessing digital health information among undergraduate students enrolled in health professional programs in Australia. Using a cross-sectional design, 610 health professional students were surveyed on their eHealth literacy. Students who were further along in their programs reported increased eHealth literacy when compared to new students, which points to the important role that curriculum plays in equipping healthcare professionals with knowledge of digital health resources. In addition, the eHealth literacy scores were higher for younger participants, those who used more digital communication platforms, and people who monitored their health digitally. In their research, Mather and colleagues contribute to health literacy and the social context which examines the *aggregate* perceived health literacy of aspiring health professionals. However, this research rests on the assumption that patients’ health literacy will likely be affected by the health literacy of their providers, who are utilized as resources. As such, the aggregate, association, and resource research traditions are closely interlinked.

Both Agner et al. (2023) [39] and Li et al. (2022) [45] examined the ways in which relationships were associated with individual health literacy. Agner et al. (2023) [39] explored how health literacy and access to health discussion partners was associated with health outcomes among Clubhouse members in Hawai’i. Like people with intellectual disabilities, individuals with serious mental illnesses (SMIs) are a uniquely vulnerable population who frequently report lower levels of health literacy than the general population [51,52], and they experience high incidence of chronic illness and early mortality [11]. They are also more likely to have compromised or limited social networks and experience social isolation [53,54]. Thus, this study examined the ways in which social network supports in psychosocial rehabilitation centers (called mental health Clubhouses [55,56] were associated with individual health literacy.

One-hundred and sixty-three members diagnosed with serious mental illnesses across nine Clubhouses were surveyed on their self-reported stigma, health literacy, mental and physical health, and social networks. The study on social networks specifically addressed health discussion partners and asked about members, staff, and relationships outside the Clubhouse. Researchers found that having a higher number of staff, such as health discussion partners, and higher levels of education were associated with needing less help in reading health-related instructions or materials. Other factors, such as an older age, male gender, and being Native Hawaiian and/or Pacific Islander, were associated with having less confidence in filling out medical forms. The study illustrates how individual and social factors intersect to affect health literacy in individuals with SMIs. Furthermore, it illustrates how community-based mental health settings that are designed to offer social support, such as Clubhouses, can play a role in supporting health literacy. Staff health discussion partners may provide natural supports for members as they navigate complex health systems, and community settings may provide alternative settings to design targeted health literacy interventions for this population.

Li et al. (2022) [45] explored how personal relationships are associated with health literacy, and they also focused on the influence of mass media and its intersection with individual vulnerabilities. Li and colleagues surveyed 812 urban older adults on their self-reported health literacy, health information sources, and personal factors that may influence health literacy, such as education, minority status, chronic illness, and age. They found that personal sources of information, such as neighbors or healthcare practitioners, had the strongest influence on health literacy and could encourage health behavior changes. Mass media, such as newspapers and television, also played a large role in influencing health knowledge through providing information about health resources and increasing reading comprehension related to health issues. This points to the importance of accessibility and affordability regarding mass media, and how barriers to access it can negatively impact health literacy, especially in older adult populations. Age was found to be negatively associated with health literacy, which was potentially due to cognitive decline and decreased access to health information, such as decreased access to digital sources of health information. Other factors, such as minority status, financial strain, low education levels, and chronic disease were also negatively associated with health literacy. These findings illustrate the importance of multiple types of relationships (neighbors and health providers) in accessing health information and the implications for considering socioeconomic factors in relation to accessing health information.

Two other articles in this Special Issue link socioeconomic class and financial resources with health literacy. Jenkins et al. (2022) [43] contributed to the *resource* research tradition approach to analyze the potential use of public libraries in England to support the development of critical health literacy in children. Interviews were conducted with 13 children, 13 public library staff, and six community stakeholders to determine how public libraries could aid in the development of health literacy. Eight child advisors were consulted in the development of the study design to ensure the research aims were relevant to the study population. Using methods from institutional ethnography, the researchers collected data through semi-structured interviews with library staff, interviews with children through child generated drawings, and analysis of library resources and supports collected during site visits. The results showed that public libraries were not seen as a setting in which people could gain health knowledge, but rather as a setting that provided resources to guide people towards where to access health information. Additionally, libraries were found to be limited in their offerings because of legislation and political considerations determining what is deemed appropriate as library-based activities. As a result, a macro-level approach to addressing health literacy could be difficult, but on the micro level, individual library health promotion efforts can successfully foster supportive environments to increase critical health literacy. Jenkins et al. suggested an approach that involves multiple settings carrying out efforts to increase children’s health literacy, such as partnerships between public libraries, schools, and other settings. As public libraries are accessible to everyone irrespective of class, and are frequented by a broad diversity of ages and individuals, this is a social context that could potentially be leveraged to overcome some disparities in accessing information.

Another study that examined the role of resources associated with socioeconomic class was conducted by Achstetter et al. (2022) [40]. They explored data on the health literacy of private health insurance insureds in Germany. In this quantitative cross-sectional study, 3601 private health insureds were surveyed on their assessments of the health system according to their health literacy levels. The results showed that almost half of the respondents had low health literacy (46.2%), and this finding was more commonly reported among men and in individuals with low subjective social statuses. Individuals with lower health literacy reported decreased satisfaction with the German health system when compared to individuals with higher health literacy. Additionally, lower health literacy was associated with greater financial burden, more experiences of discrimination during healthcare encounters, and greater inefficiencies or safety concerns, such as receiving the wrong medication or receiving unnecessary healthcare services. The results imply the importance of health literacy in receiving increased care and having fewer unmet needs and greater satisfaction with the healthcare system overall. Strengthening people’s health literacy is important to improve their access to healthcare and resources and to decrease disparities in treatment.

Finally, two publications in this Special Issue focus on organizational health literacy (OHL), which addresses system-wide strategies to support patients’ health literacy and contributes to the *distributed* research tradition, wherein health literacy is understood on a collective level rather than an individual level. Examples of OHL include organization-wide changes, such as avoiding the use of medical jargon or using visualizations to enhance patients’ understanding of health information. OHL considers the healthcare system, or organization, as a social contextual setting in which health information is shared and accessed. Stuermer et al. (2022) [48] assessed use and perceptions of an organizational health literacy assessment tool (OHL Self-AsseT), which was developed to assess and improve OHL in primary care organizations. The OHL Self-AsseT tool includes three modules: the first includes a manual with information about the concept of health literacy, the second comprises a checklist for assessing the dimensions of health literacy and the degree of fulfillment in the organization, and the third provides a handbook that assists in providing concrete strategies and actions to improve organizational health literacy.

Stuermer and colleagues gathered the attitudes and experiences of two primary care teams in Switzerland using the OHL Self-AsseT tool. Interviews were conducted with 19 healthcare professionals pre- and post-intervention to determine any changes or improvements made, and a reflexive thematic analysis was used to identify themes based on a constructivist orientation. The participants found the OHL Self-AsseT tool to be effective in offering practical strategies to increase organizational health literacy. The intervention showed an increase in momentum for change, allowing participants to act upon improvements they had not yet been able to implement and brainstorm ways to improve OHL in the future. Putting these changes into action built a sense of teamwork and collective efficacy among the providers as they were empowered to share their ideas and see the intervention strategies being carried out. This showed that OHL intervention strategies that are supportive, collaborative, and promote team building are effective in empowering healthcare professionals by giving them a sense of ownership and responsibility for implementing change and improving quality of care.

Beese et al. (2022) [41] also focused on Swiss primary care provider teams and utilized the OHL Self-AsseT tool among 10 healthcare teams (four general practitioners’ practices and six home care service organizations). However, they also examined individual health literacy among 47 primary care providers. They found that most, but not all, health providers were familiar with the concept of health literacy, and that provider teams were able to critique the organizational health literacy of their settings and come up with actionable strategies to improve it. This suggests that health professionals who are familiar with the concept will likely be important sources of information for how and whether health settings can be improved on a macro level to support patients’ ability to access and understand health information.

Together, these studies are linked with a wide variety of research traditions on health literacy and social contexts, and the conceptual overlap in their findings provides guidance towards practical implications for health professionals and researchers alike.

## 3. Practical Implications for Health Professionals and Suggestions for Future Research

Although this Special Issue included research from diverse countries, populations, and authorship teams, there are themes in this work that link with past research traditions in this area, but also point to future directions for health professionals and researchers. Broadly, we identified four themes, outlined below, with suggestions for health professionals and researchers.

### 3.1. Multiple Individual Factors and Levels of Social Context Intersect to Influence Health Literacy. A Comprehensive, Multi-Level Intervention Framework Could Guide Practice and Research

The research in this Special Issue focused primarily on social context, but the social context was defined in multiple ways and had multiple levels, and almost all studies included individual-level factors as well. This suggests that health literacy interventions can take ecological and intersectional approaches and should ask patients or target populations about who they rely on for health information and health discussion support. While intersectional and ecological approaches are gaining momentum theoretically and conceptually [57] much work remains to link those theories with quantitative methods that address co-occurring multi-level factors [58,59]. With an intersectional, ecological approach, individual and system-level interventions will likely have greater contextual validity. From the research perspective, a comprehensive, integrated theoretical framework that identifies the multiple layers of social context influencing health literacy would be a welcome addition. While we chose to link articles in this Special Issue with existing research traditions identified by Pitt et al. (2019) [50], those traditions describe the state of existing scholarship and do not serve as a guide for clinicians or practitioners to understand the dynamic and multi-layered social context within which health literacy is actualized, or how those layers can best be captured in research.

### 3.2. Social Resources and Contexts Outside the Medical Sphere Can Be Leveraged to Enhance Health Literacy in Vulnerable Populations through Meaningful Partnerships

The research in this Special Issue indicated that multiple levels of social context can be targeted for health literacy interventions. Community-based mental health centers or psychosocial rehabilitation centers can be utilized to support a vulnerable population at high risk through existing trusting relationships. Public libraries can partner with other settings, such as schools or digital spaces, to address health literacy on a macro level [31]. This may be particularly effective among populations that are disenfranchised from conventional health systems or that underutilize health systems because of cost, stigma, distance, or other factors [60,61,62]. Future research should examine best practices for developing partnerships between health professionals and trusted community sites or resources, particularly to enhance access for vulnerable populations that have had poor health outcomes, or populations (such as individuals with profound intellectual disabilities) who may require unique communication strategies that some health providers are unaware of or not experienced in.

### 3.3. Digital Interaction Spaces and Online Communication Are Central in Health Decision Making and Should Be a Focus of Health Literacy Interventions and Research

Several articles in this Special Issue identified the important and growing roles that digital interaction, social media, and mass media have on individual and collective health literacy [63]. Health professionals not only need to be trained to critically appraise online health information, but they also need to be trained in strategies to educate their patients to effectively seek and vet online health information. There are various models of digital health literacy. One of the best known is that of Norman and Skinner (2006) [64]. Digital health literacy is understood as the ability to search for, find, understand, and evaluate health information based on digital sources and to apply the knowledge gained to address health challenges and solve problems. Given our shift to primarily digital sources of information and the increasing importance of digital interaction, it is important that health providers are prepared for their patients to have strong preconceived notions from outside sources perceived as trustworthy, which may or may not be accurate [65]. Providers must not only be prepared to educate their patients and vet online health information themselves, but to negotiate the value of health information found by patients outside of clinical contexts [66,67]. Furthermore, clinicians may benefit from educating patients on appraise digital information, particularly from sources they trust. Research on best practices for navigating the intersection of online health information, patient preferences and beliefs, and the best available evidence is an important applied area as digital interaction and health information are increasingly embedded in daily life. This may also be an important consideration for policy makers, as online health information is not tightly controlled [68], and misinformation can have dire health consequences on the individual and community levels.

### 3.4. Health Literacy Has Bi-Directional Influences on Overall Community Health

As we have described, social context has a significant impact on individual health literacy. However, the strengthening of health literacy also has a reciprocal, bi-directional influence on community health. The World Health Organization (WHO) formulated the following definition in 1998 [69]: “Health literacy implies the achievement of a level of knowledge, personal skills and confidence to take action to improve personal and community health by changing personal lifestyles and living conditions”. The WHO presumes that “improving health literacy in populations provides the foundation on which citizens are enabled to play an active role in improving their own health, engage successfully with community action for health, and push governments to meet their responsibilities in addressing health and health equity” (2023) [70]. Paakkari and Paakkari (2012) [71] go so far as to view citizenship as a significant element of health literacy. They argue that this includes the ability to look beyond one’s own perspective, to include the perspective of others up to a group or collective, and thus, to not only adapt one’s own behavior but also affect changes, e.g., on an organizational level. Future research should focus on the reciprocal process of strengthening health literacy, such as the influence of organizational health literacy on different involved groups of individuals [72].

## 4. Conclusions

This editorial situates the articles in this Special Issue within existing research traditions on health literacy in the social context and provides an opportunity to identify fruitful future directions for research, theory, and practice. As outlined in the anecdote that introduced this Special Issue, we frequently rely on, and are influenced by, a wide range of intersecting social factors and contexts when seeking health information and making health decisions. Future work should expand the use of social contexts to strengthen people’s understanding of crucial health information and to support populations that suffer health disparities, thereby reducing systematic disparities in health [73]. Furthermore, we must also recognize and consider the ways in which social contexts can create barriers to health literacy. For example, misinformation can be spread through trusted social networks, and organizational health literacy may be halted by administrators or healthcare settings resistant to change. Moving research and multi-level interventions forward in this area will not only require creativity, but also a willingness to adapt to the fluid and multifaceted environmental influences that shape our ability to access, assess, and utilize information to improve health.

## Figures and Tables

**Table 1:** Health literacy and social context special issue article characteristics.

Citation	N	Country	Population	Setting	Aim/Purpose
[39]	163	USA	People with serious mental illnesses attending Clubhouses	Mental health center	Examines the relationship between health literacy, social networks, self-efficacy, self-rated health, and stigma among people with serious mental illness in Hawai’i
[40]	3601	Germany	People with private health insurance	Healthcare	Examines “the health literacy of private health insurance insureds in Germany and analyze their assessment of the health system according to their health literacy level” (p. 1)
[41]	74	Switzerland	Primary care teams and healthcare professionals	Healthcare	“Assess organizational health literacy (OHL) in Swiss primary care organizations” (p. 1)
[42]	125	Germany	Disability care professionals and people with profound intellectual disabilities	Intellectual disabilities	Addresses the gap in approaches to communicate health-related needs and questions for people with profound intellectual disabilities
[43]	32	England	Children and public library staff	Public library	“Analyzes the potential of public libraries in England to be supportive environments for children’s development of critical health literacy” (p. 1)
[44]	21	Norway	Patients and providers	Healthcare	Summarizes existing studies examining patient and provider communication about online health information
[45]	812	China	Urban citizens aged 60 and older	Community	Explores “the link between health information sources and health literacy” among older adults (p. 1)
[46]	610	Australia	Undergraduate health profession students	University	Analyzes the online health literacy of Australian “health profession students to inform undergraduate curriculum development and promote work-readiness” (p. 1)
[47]	324	USA	Residents of Hawai’i aged 18–35	Community	Explores “social network variation & health information sharing during COVID-19, especially for Native Hawaiians, other Pacific Islanders, and Filipinos who experienced COVID-19 inequities” (p. 1)
[48]	19	Switzerland	Primary care teams and healthcare professionals	Healthcare	Explores how an organizational health literacy assessment tool (OHL Self-AsseT) was implemented by primary care teams in Switzerland
[49]	38	Germany	People with mild to moderate intellectual disabilities	Community	Explores which dimensions influence the health literacy of people with intellectual disabilities
